# Supplementary material for: Small tandem DNA duplications result from CST-guided Pol α-primase action at DNA break termini
Source: Nat Commun. 2021 Aug 10;12:4843. doi: 10.1038/s41467-021-25154-w (PMC8355091; doi:10.1038/s41467-021-25154-w)
Supplement: Supplementary file 1 — Supplementary Information [file 41467_2021_25154_MOESM1_ESM.pdf]

Supplemental information

Small tandem DNA duplications result from CST-guided Pol α-primase action at DNA break termini

Joost Schimmel, Núria Muñoz-Subirana, Hanneke Kool, Robin van Schendel and Marcel Tijsterman

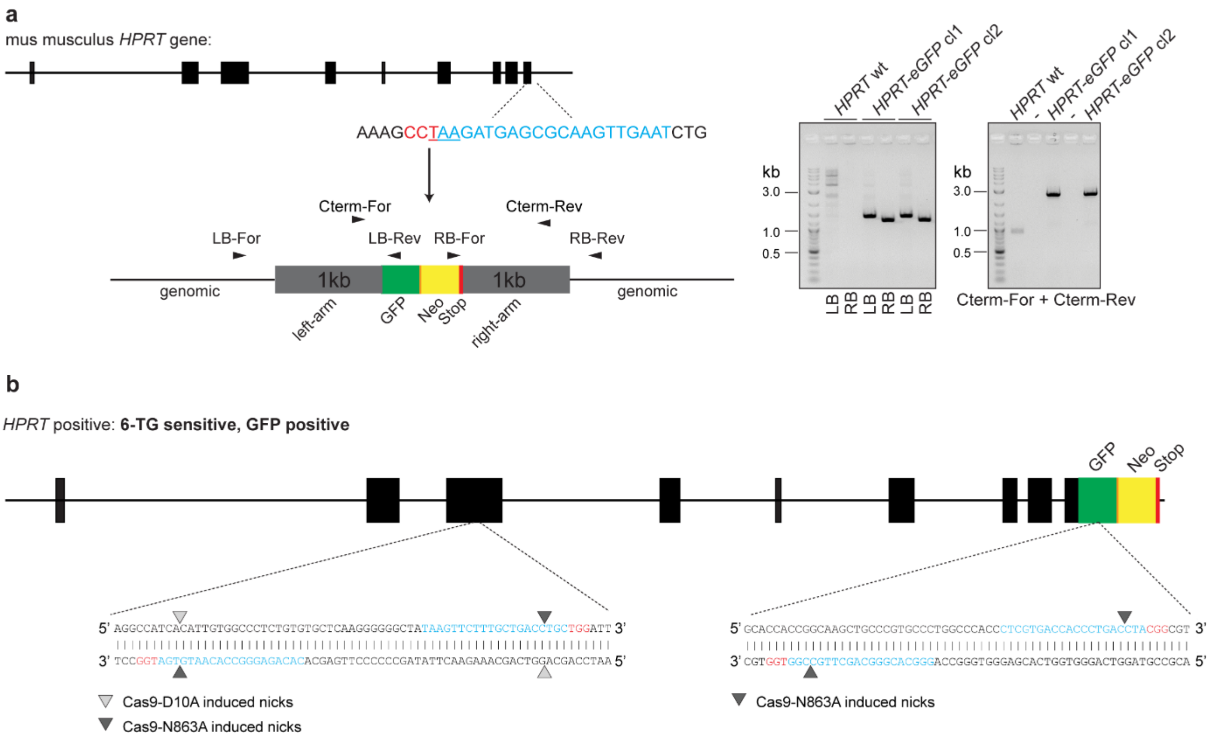

Supplementary Figure 1: Generation and validation of HPRT-eGFP reporter cell-lines

a, Targeting strategy to generate endogenous HPRT-eGFP knockin. Left, schematic representation of the HPRT gene and the sgRNA (cyan) and PAM-site (red) used to target the stop-codon (underlined sequence). Successful targeting using a construct with 1kb HPRT homology-arms will result in a HPRT-eGFP-P2A-Neomycin-Stop product. Right, DNA-gels showing the confirmation of correct targeting in two independent GFP-positive wild-type mES clones (cl1 and cl2). PCRs were performed with the indicated primers for the left border (LB), the right border (RB) (left panel) or with Cterm-For and Cterm-Rev (right panel). Non-targeted wild-type mES cells (HPRT wt) are used as a negative control. Representative example of at least two independent PCRs is shown. **b**, Schematic showing the HPRT-eGFP gene (top) and the sequences of the Cas9-target sites (bottom) used in this study (in exon 3 and eGFP). sgRNAs are indicated in cyan, PAM-sites in red. Theoretical sites of nicking by Cas9-N863A or Cas9-D10A are indicated by triangles.

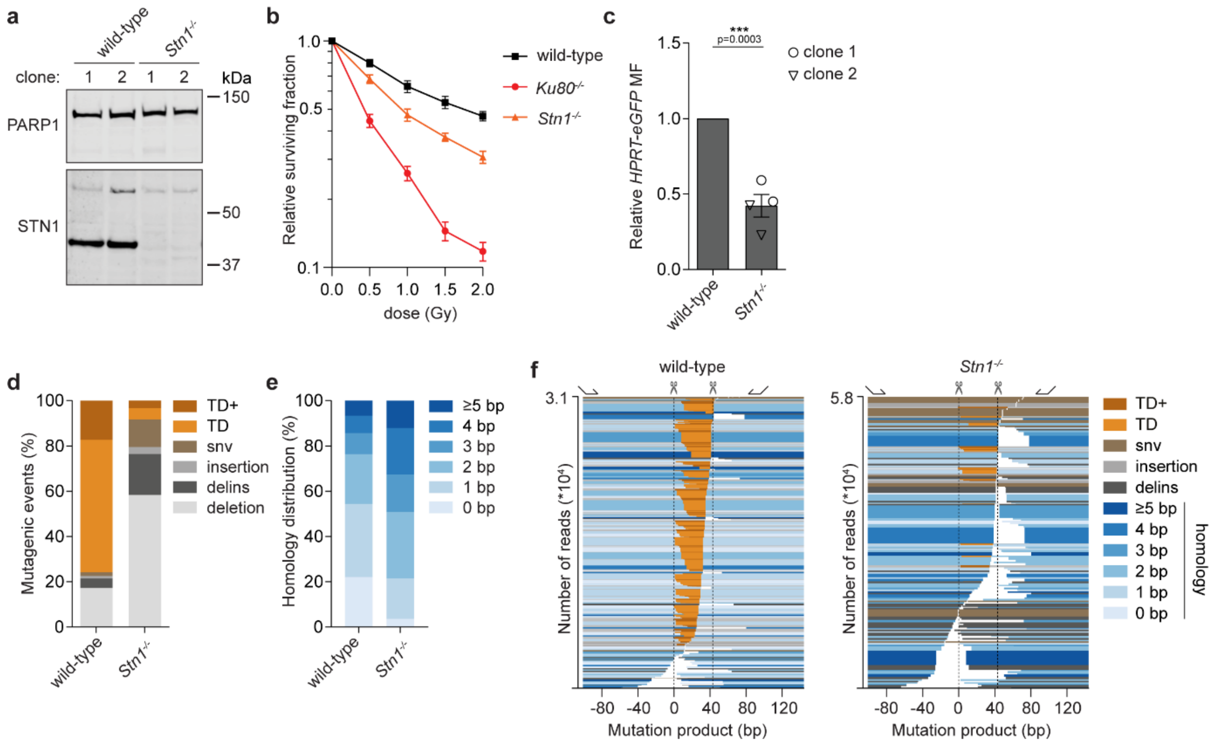

Supplementary Figure 2: Tandem duplication formation at 3' ssDNA requires Stn1

a, Immunoblot confirming loss of STN1 protein expression in two independent Stn1 knockout clones (lower panel). An immunoblot for PARP1 is included as a loading control (upper panel). Representative example of at least two independent immunoblots is shown. **b**, Clonogenic survival of wild-type, Stn1$^{-/-}$ and Ku80$^{-/-}$ mES cell-lines after exposure to different doses of ionizing radiation. Data shown are the mean ± SEM (n = 8, two experiments using two independent clones per genotype done in duplicate). **c**, Relative *HPRT-eGFP* mutation frequencies (MF) of breaks with 3' ssDNA overhangs for wild-type and Stn1$^{-/-}$ mES cell-lines (using two independent clones per genotype) transfected with Cas9-N863A and sgRNAs targeting eGFP. The data shown represent the mean ± SEM (n = 4) and are expressed as a fraction of the mutation frequency observed in wild-type cells (set to 1). Statistical significance was calculated via two-tailed unpaired t-test. ***P ≤ 0.001. **d**, Quantification of the types of mutagenic events (see methods for details) at breaks with 3' ssDNA overhangs for wild-type and Stn1$^{-/-}$ mES cells transfected with Cas9-N863A and sgRNAs targeting eGFP. GFP-negative (mutant) cells were sorted and used for targeted sequencing around the break site. **e**, Quantification of the extent of microhomology used for deletions and tandem duplications (TD) in the indicated genotypes. **f**,

Mutational signatures from **d** plotted as bars, relative to the Cas9-N863A induced nicks (dashed lines). The degree of blue colouring reflects the extent of microhomology. For TDs the duplicated sequence is plotted in orange. The height of each bar reflects the contribution of each outcome in the total amount of reads (Y-axis). Delins, deletion insertion; snv, single nucleotide variant; TD, tandem duplication; TD+, tandem duplication with additional mutation (see Methods); bp, base pair.

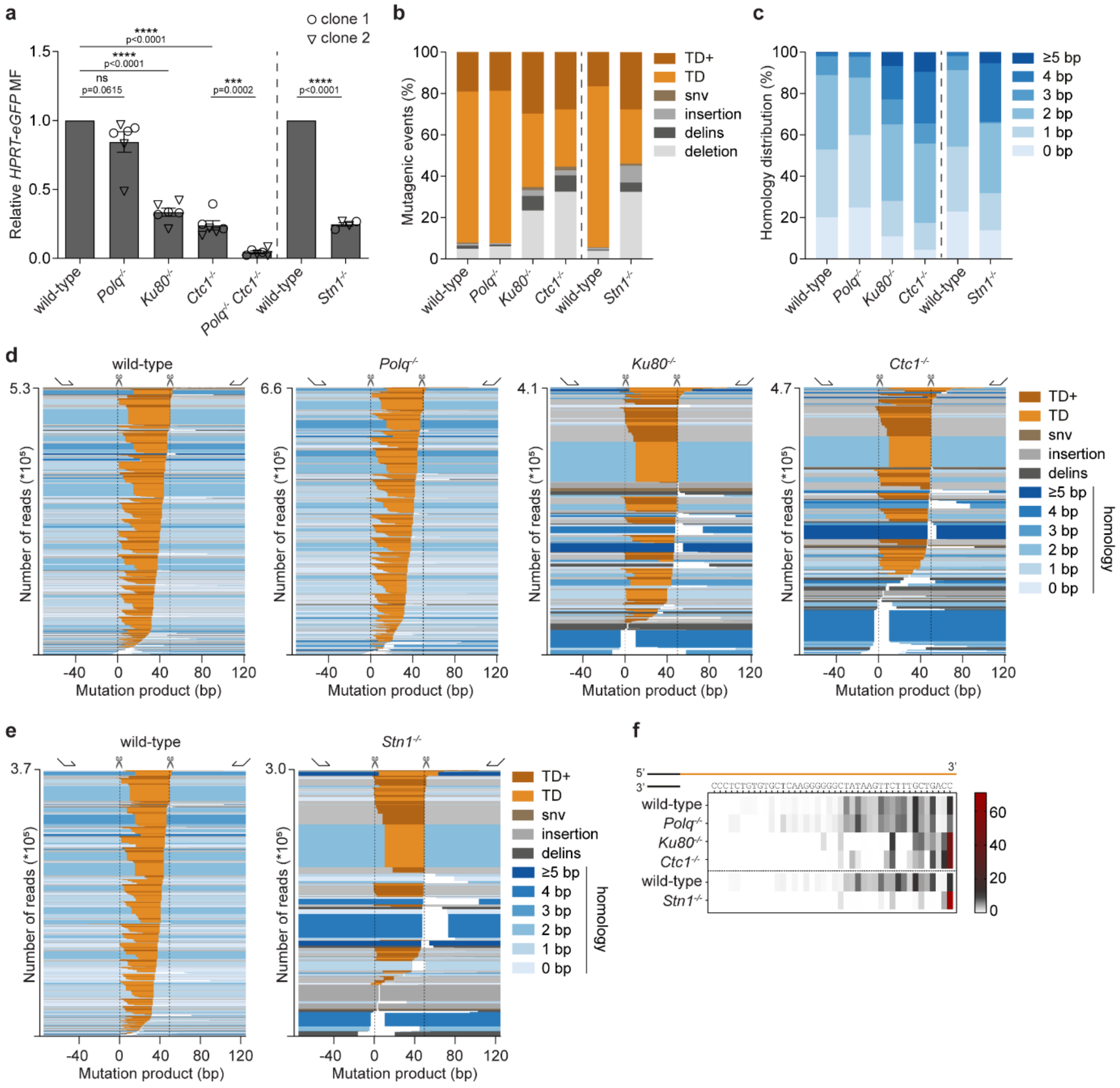

Supplementary Figure 3: CST-complex promotes tandem duplication formation on small 3' ssDNA overhangs at HPRT exon 3.

a, Relative *HPRT-eGFP* mutation frequency (MF) of breaks with 3' ssDNA overhangs for the indicated mES cell-lines (using two independent clones per genotype) transfected with Cas9-N863A and sgRNAs targeting HPRT exon 3. The data shown represent the mean ± SEM (n = 6 for wild-type versus Polq$^{-/-}$, Ku80$^{-/-}$ and Ctc1$^{-/-}$ mES cells; n= 4 for wild-type versus Stn1$^{-/-}$ cells) and are expressed as a fraction of the mutation frequency observed in wild-type cells (set to 1). Statistical significance was calculated via two-tailed unpaired t-test. ns, not significant (P > 0.05), ***P ≤ 0.001 ****P ≤ 0.0001. **b**, Quantification of the types of mutagenic events (see methods for details) at breaks with 3' ssDNA overhangs for the

indicated mES cell-lines transfected with Cas9-N863A and sgRNAs targeting HPRT exon 3. GFP-negative (mutant) cells were sorted and used for targeted sequencing around the break-site, data represent the average of two independent clones for wild-type, Polq$^{-/-}$, Ku80$^{-/-}$ and Ctc1$^{-/-}$ mES cells (left) and for one wild-type and Stn1$^{-/-}$ cell line (right). **c**, Quantification of the extent of microhomology used for deletions and tandem duplications (TD) in the indicated genotypes. **d** and **e**, Mutational signatures from **b** plotted as bars, relative to the Cas9-N863A induced nicks (dashed lines). The degree of blue colouring reflects the extent of microhomology. For TDs the duplicated sequence is plotted in orange. The height of each bar reflects the contribution of each outcome in the total amount of reads (Y-axis). **f**, Heat-maps representing the relative start position of the TDs from **b** on the left 3' overhang of Cas9-N863A induced DSBs. Data is shown as percentage of the total amount of TDs in the spectra. Delins, deletion insertion; snv, single nucleotide variant; TD, tandem duplication; TD+, tandem duplication with additional mutation (see Methods); bp, base pair.

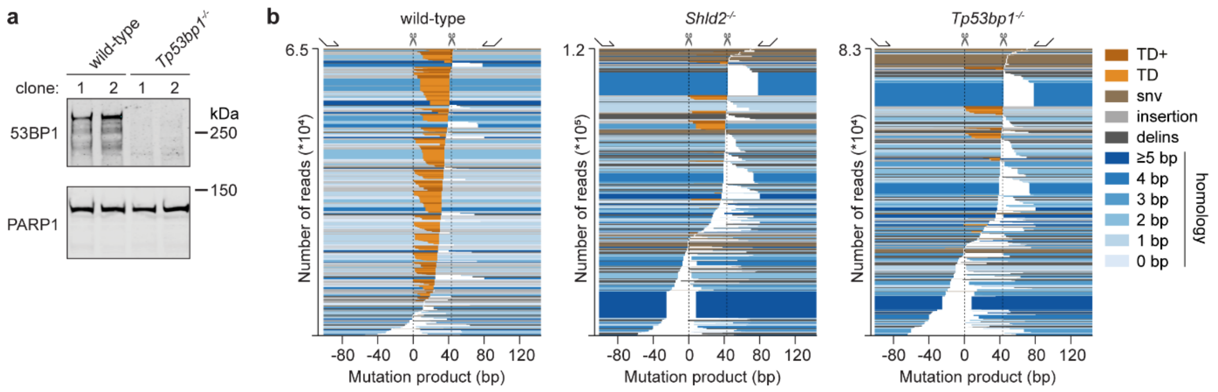

Supplementary Figure 4: Tandem duplication formation at 3' ssDNA in mouse cells requires the 53BP1-Shieldin axis.

a, Immunoblot confirming loss of 53BP1 protein expression in two independent Tp53bp1 knockout clones (upper panel). An immunoblot for PARP1 is included as a loading control (lower panel). Representative example of at least two independent immunoblots is shown. **b**, Mutational signatures from Fig. 3b plotted as bars, relative to the Cas9-N863A induced nicks (dashed lines) in eGFP. The degree of blue colouring reflects the extent of microhomology and TDs are plotted in orange, representing their origin. The height of each bar represents its contribution to the total amount of reads (Y-axis).

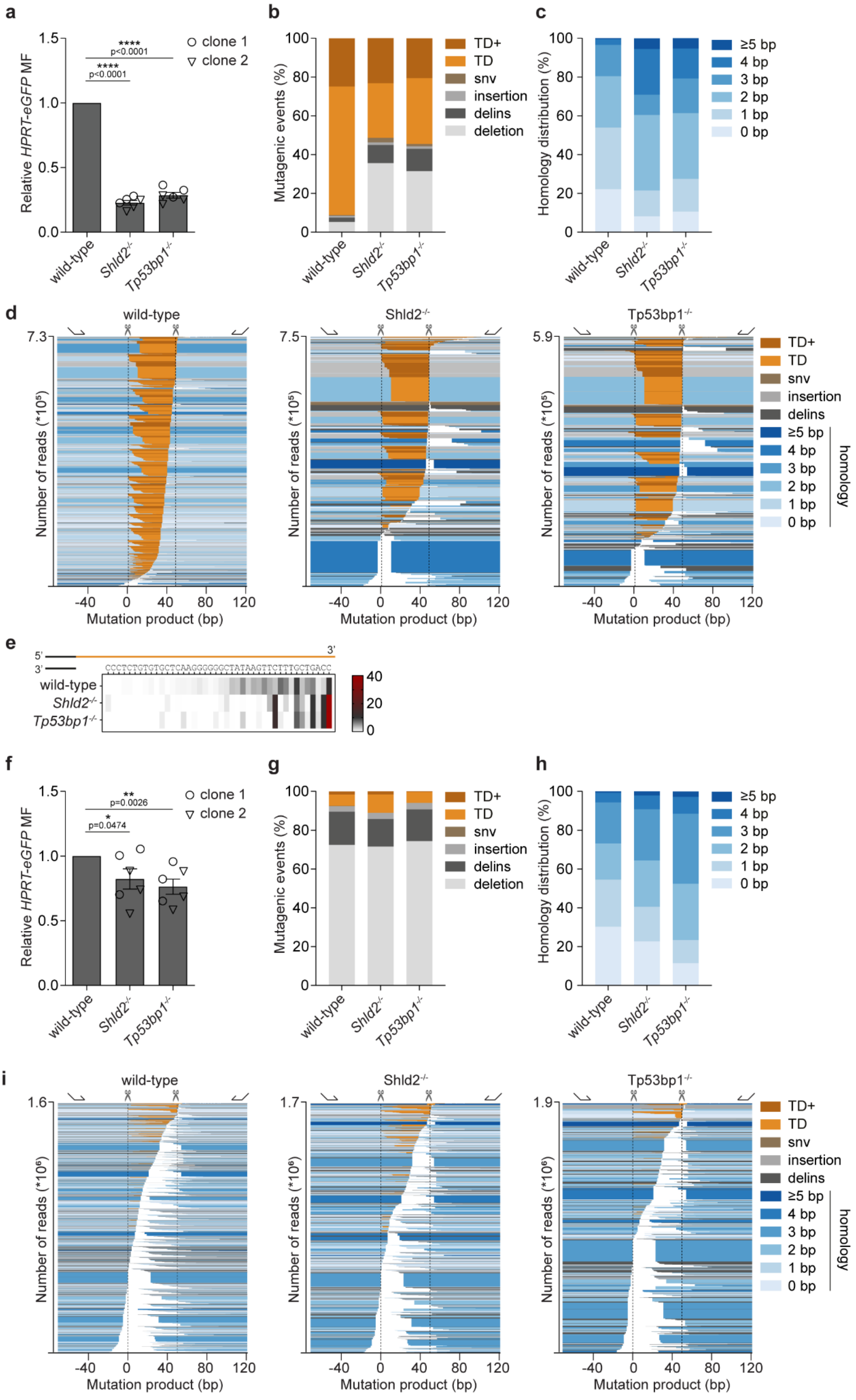

Figure S5 *(legend on next page)*.

Supplementary Figure 5: 53BP1 and Shieldin promotes tandem duplication formation specifically on 3’ ssDNA overhangs.

a, Relative *HPRT-eGFP* mutation frequency (MF) for the indicated mES cell-lines (using two independent clones per genotype) transfected with Cas9-N863A and sgRNAs targeting HPRT exon 3, producing DSBs with 3’ ssDNA overhangs. The data shown represent the mean ± SEM (n = 6) and are expressed as a fraction of the mutation frequency observed in wild-type cells (set to 1). Statistical significance was calculated via two-tailed unpaired t-test. ****P ≤ 0.0001. **b**, Quantification of the types of mutagenic events (see methods for details) at breaks with 3’ ssDNA overhangs for the indicated mES cell-lines transfected with Cas9-N863A and sgRNAs targeting HPRT exon 3. 6-TG resistant (mutant) cells were selected and used for targeted sequencing around the break-site, data represent the average of two independent clones. **c**, Quantification of the extent of microhomology used for deletions and tandem duplications (TD) in the indicated genotypes. **d**, Mutational signatures from **b** plotted as bars, relative to the Cas9-N863A induced nicks (dashed lines). The degree of blue colouring reflects the extent of microhomology. For TDs the duplicated sequence is plotted in orange. The height of each bar reflects the contribution of each outcome in the total amount of reads (Y-axis). **e**, Heat-map representing the relative start position of the TDs from **b** on the left 3’ overhang of Cas9-N863A induced DSBs. Data is shown as percentage of the total amount of TDs in the spectra. **f**, Relative *HPRT-eGFP* mutation frequency (MF) for the indicated mES cell-lines (using two independent clones per genotype) transfected with Cas9-D10A and sgRNAs targeting HPRT exon 3, producing DSBs with 5’ ssDNA overhangs. The data shown represent the mean ± SEM (n = 6) and are expressed as a fraction of the mutation frequency observed in wild-type cells (set to 1). Statistical significance was calculated via two-tailed unpaired t-test. *P ≤ 0.05, **P ≤ 0.01. **g**, Quantification of the types of mutagenic events (see methods for details) at breaks with 5’ ssDNA overhangs for the indicated mES cell-lines transfected with Cas9-D10A and sgRNAs targeting HPRT exon 3. 6-tg resistant (mutant) cells were selected and used for targeted sequencing around the break-site, data represent the average of two independent clones. **h**, Quantification of the extent of microhomology used for deletions and tandem

duplications (TD) in the indicated genotypes. **i**, Mutational signatures from **g** plotted as bars, relative to the Cas9-D10A induced nicks (dashed lines). The degree of blue colouring reflects the extent of microhomology. For TDs the duplicated sequence is plotted in orange. The height of each bar reflects the contribution of each outcome in the total amount of reads (Y-axis). Delins, deletion insertion; snv, single nucleotide variant; TD, tandem duplication; TD+, tandem duplication with additional mutation (see Methods); bp, base pair.

a

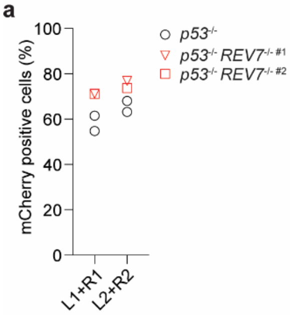

b

	L1 + R1		L2 + R2	
**type**	***p53$^{-/-}$***	***p53$^{-/-}$ REV7$^{-/-}$***	***p53$^{-/-}$***	***p53$^{-/-}$ REV7$^{-/-}$***
**deletion**	2.80%	1.46%	2.52%	0.74%
**delins**	0.58%	0.39%	0.69%	0.27%
**insertion**	1.99%	1.91%	0.34%	0.20%
**snv**	0.82%	0.95%	0.97%	1.10%
**TD**	2.81%	0.10%	7.16%	0.31%
**TD+**	0.44%	0.07%	3.01%	0.13%
**wild-type**	90.55%	95.11%	85.31%	97.26%
**Total**	100.00%	100.00%	100.00%	100.00%
**TD total**	3.25%	0.17%	10.17%	0.44%

c

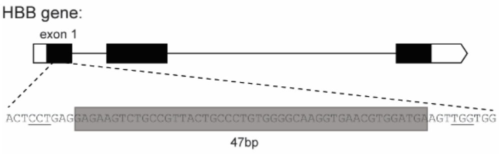

d

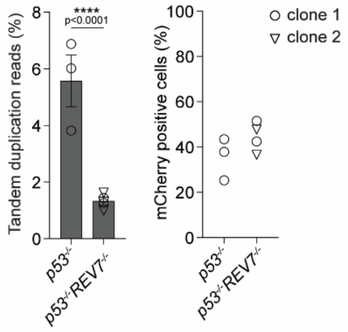

e

	HBB	
**type**	***p53$^{-/-}$***	***p53$^{-/-}$ REV7$^{-/-}$***
**deletion**	0.48%	0.55%
**delins**	0.28%	0.29%
**insertion**	0.04%	0.02%
**snv**	1.43%	1.76%
**TD**	3.98%	1.07%
**TD+**	1.59%	0.26%
**wild-type**	92.20%	96.04%
**Total**	100.00%	100.00%
**TD total**	5.57%	1.33%

f

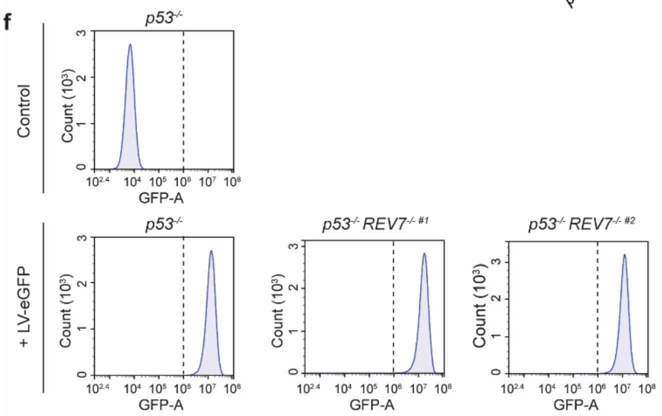

g

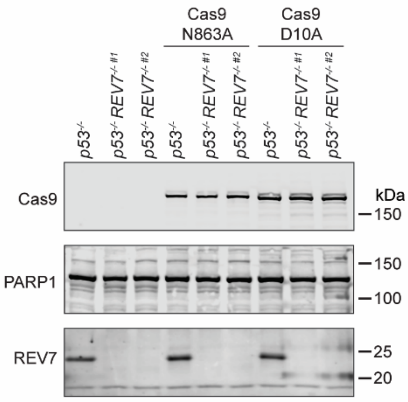

h

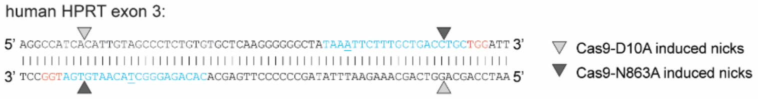

i

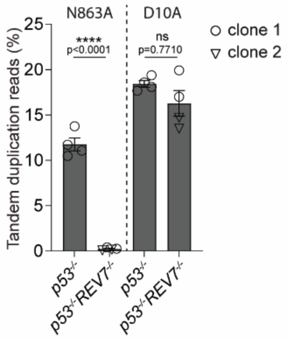

j

	N863A		D10A	
**type**	***p53$^{-/-}$***	***p53$^{-/-}$ REV7$^{-/-}$***	***p53$^{-/-}$***	***p53$^{-/-}$ REV7$^{-/-}$***
**deletion**	0.35%	0.50%	23.34%	20.56%
**delins**	0.16%	0.19%	8.16%	6.59%
**insertion**	0.06%	0.01%	1.12%	0.99%
**snv**	0.87%	1.14%	0.37%	0.47%
**TD**	10.35%	0.14%	15.61%	13.68%
**TD+**	1.40%	0.08%	2.81%	2.59%
**wild-type**	86.81%	97.93%	48.58%	55.12%
**Total**	100.00%	100.00%	100.00%	100.00%
**TD total**	11.75%	0.22%	18.42%	16.27%

Figure S6 *(legend on next page)*.

Supplementary Figure 6: Tandem duplication formation at 3' ssDNA requires REV7 in human cells.

a, Quantification of mCherry positive cells (for the experiment in Figure 3F) two days after electroporation of RPE1-p53$^{-/-}$ or RPE1-p53$^{-/-}$ REV7$^{-/-}$ cell-lines with Cas9-N863A-P2A-mCherry and the indicated FLT3 sgRNAs. **b**, Table showing the percentage for each sequence-type in the total amount of reads obtained from indicated RPE1 cell-lines electroporated with Cas9-N863A and FLT3 sgRNAs. Data represents the average of two RPE1-p53$^{-/-}$ samples and two independent RPE1-p53$^{-/-}$ REV7$^{-/-}$ clones. **c**, Schematic representation of the target site in HBB exon 1, PAM-sites are underlined. **d**, Quantification of the number of TD and TD+ reads derived from human RPE1-p53$^{-/-}$ and RPE1-p53$^{-/-}$ REV7$^{-/-}$ cells electroporated with Cas9-N863A and the indicated sgRNAs (left) and quantification of mCherry-positive cells two days after electroporation (right). Unselected cells were used for targeted sequencing around the break-site. Data represents the average ± SEM of three RPE1-p53$^{-/-}$ samples and four RPE1-p53$^{-/-}$ REV7$^{-/-}$ samples. Statistical significance was calculated via two-way ANOVA using Sidak correction. **** $P \leq 0.0001$. **e**, Table showing the percentage for each sequence type in the total amount of reads obtained from the experiment in **d**. **f**, FACS-plots of control RPE1-p53$^{-/-}$ cells and indicated RPE1 cells stably expressing eGFP. **g**, Immunoblots confirming Cas9-N863A or Cas9-D10A expression in indicated cell-lines (upper panel). An immunoblot for PARP1 is included as a loading control (middle panel) and an immunoblot for REV7 to confirm loss of REV7 protein expression in the two knockout clones (lower panel). Representative example of at least two independent immunoblots is shown. **h**, Schematic showing the human HPRT exon 3 Cas9-target sites. sgRNAs are indicated in cyan (variants in the sgRNA sequence as compared to mouse HPRT target sites (figure S1b) are underlined), PAM-sites in red. Theoretical sites of nicking by Cas9-N863A or Cas9-D10A are indicated by triangles. **i**, Quantification of the number of TD and TD+ reads at breaks with 3' ssDNA or 5' ssDNA overhangs for the indicated RPE1 cell-lines expressing Cas9-N863A or Cas9-D10A respectively and transfected with sgRNAs targeting human HPRT exon 3. Unselected cells were used for targeted sequencing around the break-site, data represent the average ± SEM of four independent samples per genotype. Statistical significance was calculated via two-way ANOVA using Sidak correction. **** P ≤

0.0001. **j**, Table showing the percentage for each sequence-type in the total amount of reads obtained from the experiment in **i**. Delins, deletion insertion; snv, single nucleotide variant; TD, tandem duplication; TD+, tandem duplication with additional mutation (see Methods); bp, base pair.

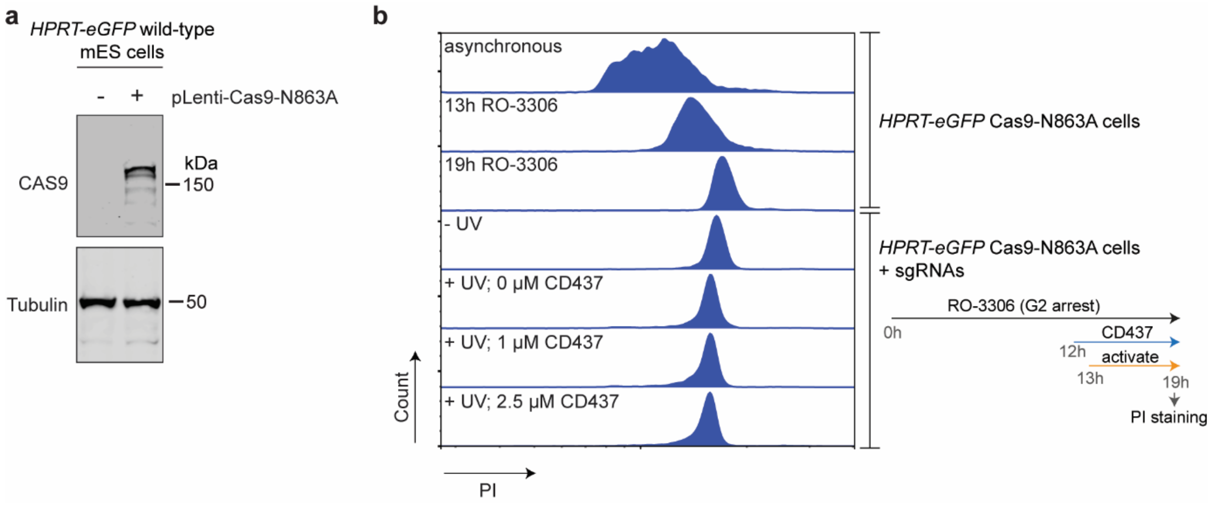

Supplementary Figure 7: Immunoblot and cell-cycle profiles light-inducible sgRNA experiment

a, Immunoblot confirming Cas9-N863A expression in HPRT-eGFP wild-type mES cells (upper panel). An immunoblot for Tubulin is included as a loading control (lower panel). Representative example of at least two independent immunoblots is shown. **b**, Quantification of Propidium-Iodide (PI) staining to analyze cell-cycle profiles of asynchronous control cells, control cells at different time points after CDK1 inhibition (RO-3306) (upper three rows), and G2/M synchronized cells used to activate the light-inducible sgRNAs in the absence or presence of Pol-alpha inhibitor (CD437) (lower four rows, corresponding to samples used in Figure 4b).

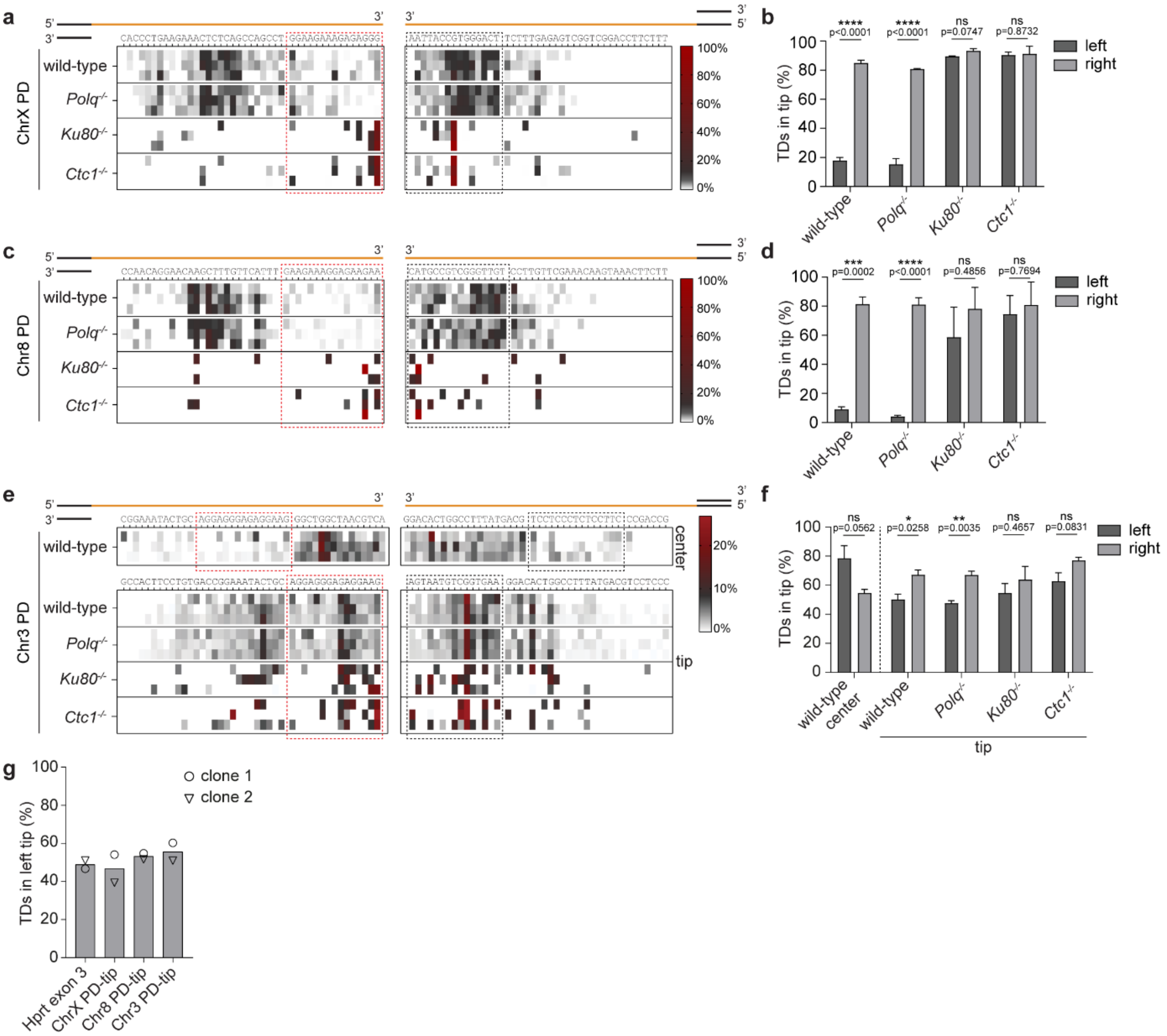

Supplementary Figure 8: Primase-desert experiments in different genetic backgrounds

a, Heat maps representing the relative start position of TDs on the left and right 3’ overhang of Cas9-N863A induced DSBs at a site containing a primase desert (PD) in the tip of the left overhang on chromosome X in the indicated genotypes. **b**, Quantification of the amount of TDs from **a** that originate from fill-in synthesis that started in the tip (first 15 nucleotides from the 3’ end) of the left and right overhang. **c**, Heat maps representing the relative start position of TDs on the left and right 3’ overhang of Cas9-N863A induced DSBs at a site containing a PD-zone in the tip of the left overhang on chromosome 8 in the indicated genotypes. NB: the wild-type data is the same data as shown in Figure 4h, Chr8 PD-tip) **d**, Quantification of the amount of TDs from **c** that originate from fill-in synthesis that started in the tip (first 16 nucleotides from the 3’ end) of the left and right overhang. **e**, Heat-maps representing the relative start position of TDs on the left and right 3’ overhang of Cas9-

N863A induced DSBs at a site containing a PD-zone in the middle of the overhang in wild-type cells (top) or at the tip of the left overhang on chromosome 3 in the indicated genotypes (bottom). **f**, Quantification of the amount of TDs from **e** that originate from fill-in synthesis that started in the tip (first 15 nucleotides from the 3' end) of the left and right overhang. Heat-maps show three individual experiments per target site in mES cell-lines of indicated genotype, and the data is shown as percentage of the total amount of TDs in the spectra. Data shown in **b**, **d** and **f** represent the mean ± SEM (n=3), statistical significance was calculated via two-tailed unpaired t-test. ns, not significant, *P ≤ 0.05, **P ≤ 0.01, ***P ≤ 0.001 ****P ≤ 0.0001. **g**, Quantification of the amount of TDs that map to outermost 15 nucleotides of the 5' protruding end of DSB induced by Cas9-D10A, either in HPRT exon 3 or in the indicated PD-tip-containing target-sites. Data represents the mean of two individual mES wild-type clones. PD, Primase desert; TD, tandem duplication.

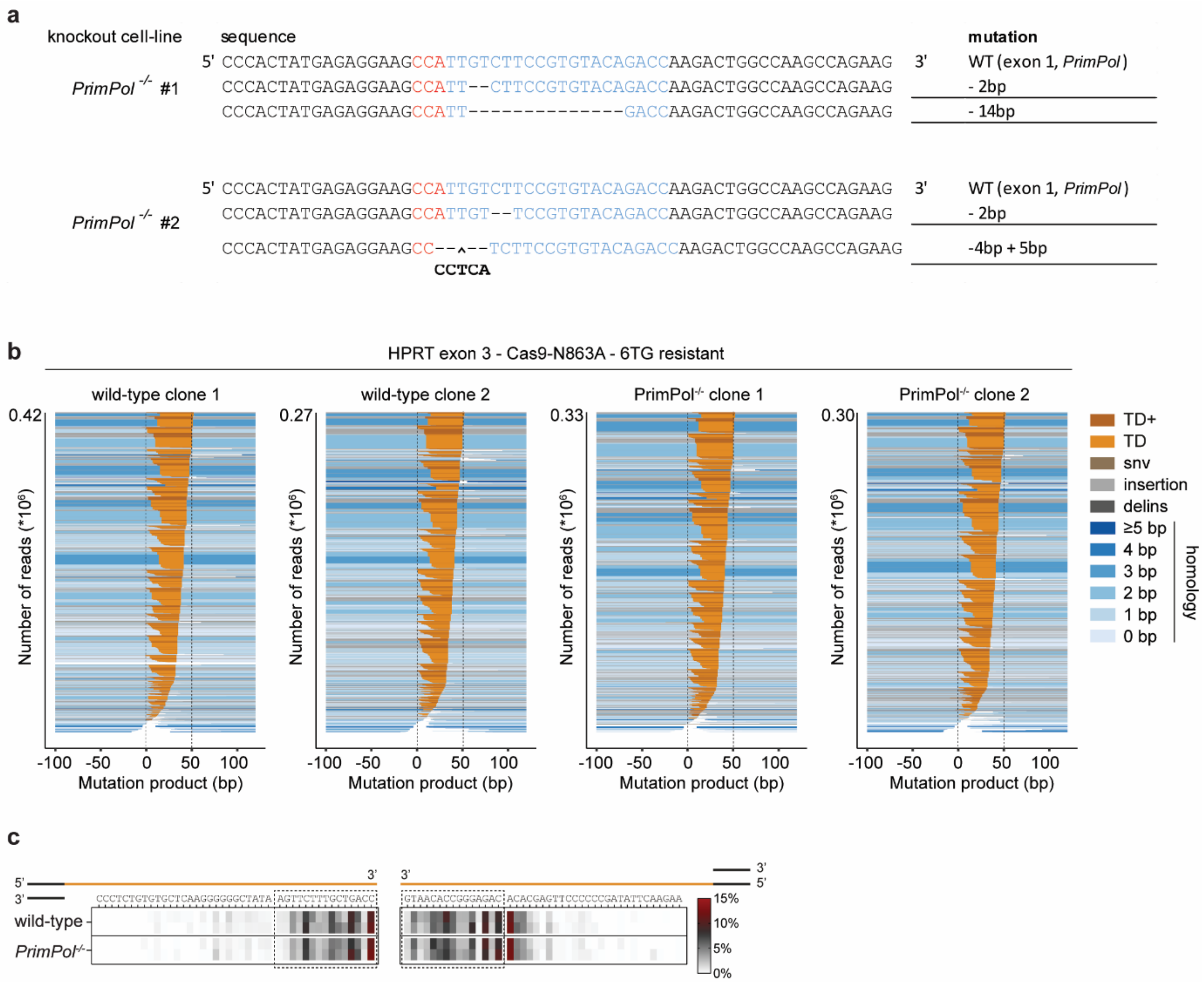

Supplementary Figure 9: PrimPol does not affect TD-formation at Cas9-N863A induced breaks with 3’ overhangs

a, sequence analysis of two PrimPol knockout clones, confirming out-of-frame mutations in exon 1. **b**, mutational signatures (see methods for details) at breaks with 3’ ssDNA overhangs for the indicated mES cell-lines transfected with Cas9-N863A and sgRNAs targeting HPRT exon 3. GFP-negative (mutant) cells were sorted and used for targeted sequencing around the break-site. **c**, Heat maps representing the relative start position of TDs at Cas9-N863A-induced DSBs with 3’ overhangs in HPRT exon 3 for wild-type and PrimPol$^{-/-}$ cells
