# Supplementary material for: Small tandem DNA duplications result from CST-guided Pol α-primase action at DNA break termini
Source: Nat Commun. 2021 Aug 10;12:4843. doi: 10.1038/s41467-021-25154-w (PMC8355091; doi:10.1038/s41467-021-25154-w)
Supplement: Supplementary file 3 — Description of Additional Supplementary Files [file 41467_2021_25154_MOESM3_ESM.pdf]

Description of Additional Supplementary Files

File Name: Supplementary Data 1
Description: overview of used mES cell-lines

File Name: Supplementary Data 2
Description: overview of Cas9-nickase target-sites

File Name: Supplementary Data 3
Description: Statistical analysis targeted sequencing

File Name: Supplementary Data 4
Description: Description raw targeted sequences-files
