# Supplementary material for: Small tandem DNA duplications result from CST-guided Pol α-primase action at DNA break termini
Source: Nat Commun. 2021 Aug 10;12:4843. doi: 10.1038/s41467-021-25154-w (PMC8355091; doi:10.1038/s41467-021-25154-w)
Supplement: Supplementary file 8 — Reporting Summary [file 41467_2021_25154_MOESM8_ESM.pdf]

nature research

Corresponding author(s): Marcel Tijsterman

Last updated by author(s): Jun 30, 2021

Reporting Summary

Nature Research wishes to improve the reproducibility of the work that we publish. This form provides structure for consistency and transparency in reporting. For further information on Nature Research policies, see our Editorial Policies and the Editorial Policy Checklist.

Statistics

For all statistical analyses, confirm that the following items are present in the figure legend, table legend, main text, or Methods section.

n/a	Confirmed	
☐	☒	The exact sample size (*n*) for each experimental group/condition, given as a discrete number and unit of measurement
☒	☐	A statement on whether measurements were taken from distinct samples or whether the same sample was measured repeatedly
☐	☒	The statistical test(s) used AND whether they are one- or two-sided<br>*Only common tests should be described solely by name; describe more complex techniques in the Methods section.*
☒	☐	A description of all covariates tested
☒	☐	A description of any assumptions or corrections, such as tests of normality and adjustment for multiple comparisons
☐	☒	A full description of the statistical parameters including central tendency (e.g. means) or other basic estimates (e.g. regression coefficient) AND variation (e.g. standard deviation) or associated estimates of uncertainty (e.g. confidence intervals)
☐	☒	For null hypothesis testing, the test statistic (e.g. *F*, *t*, *r*) with confidence intervals, effect sizes, degrees of freedom and *P* value noted<br>*Give P values as exact values whenever suitable.*
☒	☐	For Bayesian analysis, information on the choice of priors and Markov chain Monte Carlo settings
☒	☐	For hierarchical and complex designs, identification of the appropriate level for tests and full reporting of outcomes
☒	☐	Estimates of effect sizes (e.g. Cohen's *d*, Pearson's *r*), indicating how they were calculated

Our web collection on statistics for biologists contains articles on many of the points above.

Software and code

Policy information about availability of computer code

Data collection	FLASH v2.2.00 was used to merge paired-end data. Subsequent quality checking, mapping and mutation calling was performed using in-house software (manuscript in preparation, available upon request). Immunoblots were scanned with the odyssey CLx (LI-COR biosciences)
Data analysis	Graphs were plotted and analyzed using Graphpad Prism 8 (v8.4.2). Mutational signature spectra (as in Fig 1g) were generated using R (version 4.0.2). The NovoExpress (version 1.4.1) and BD FACSDiva software (version 9.0.1) was used to analyze the flow cytometry data. Immunoblots were analyzed with Image Studio (version 5.2, LI-COR biosciences).

For manuscripts utilizing custom algorithms or software that are central to the research but not yet described in published literature, software must be made available to editors and reviewers. We strongly encourage code deposition in a community repository (e.g. GitHub). See the Nature Research guidelines for submitting code & software for further information.

Data

Policy information about availability of data

All manuscripts must include a data availability statement. This statement should provide the following information, where applicable:

- Accession codes, unique identifiers, or web links for publicly available datasets
- A list of figures that have associated raw data
- A description of any restrictions on data availability

Raw targeted sequences have been made publicly available at NCBI SRA (Accession code PRJNA641538, https://www.ncbi.nlm.nih.gov/bioproject/PRJNA641538). A description of the sequence-files and matching figures can be found in Table S4. Source data are provided with this paper. FLT3-ITDs represented in Figure 3g are obtained from the publicly accessible COSMIC database (https://cancer.sanger.ac.uk/cosmic).

Field-specific reporting

Please select the one below that is the best fit for your research. If you are not sure, read the appropriate sections before making your selection.

☒ Life sciences ☐ Behavioural & social sciences ☐ Ecological, evolutionary & environmental sciences

For a reference copy of the document with all sections, see nature.com/documents/nr-reporting-summary-flat.pdf

Life sciences study design

All studies must disclose on these points even when the disclosure is negative.

Sample size	No sample-size calculation was performed. For targeted sequencing we determined the minimal amount of cells and DNA needed to obtain mutational signatures with sufficient depth and reads. Sample sizes were chosen for the different experimental approaches based on the technical difficulty and throughput of the individual assays, the chosen sample sizes are consistent with previous publications and common practices in the field (2-3 biological replicates)
Data exclusions	For the analysis of mutational signatures in selected HPRT-eGFP mutant samples, 'wild-type' reads from targeted sequencing were excluded from the analysis. This is stated in the 'Methods' section of our manuscript. No other data has been excluded
Replication	We followed common practices in the field (2-3 biological replicates with two independent derived knockout lines per genotype). Mutation frequency was determined by performing at least two replicates with two independent clones per genotype. Mutational signatures were also derived from two independent derived clones or biological replicates.<br>All replication attempts were successful, the number of replicate experiments are indicated in the figure legends of the manuscript.
Randomization	Allocation was not randomized. Covariates were controlled by taking along parental cell-lines in each experiment
Blinding	Investigators were not blinded during the study. Numbering of biological samples was used during experiments, but the investigators made the numbering themselves. Data analyses were performed by unbiased software programs/algorithms, blinding was therefore not applicable to our study

Reporting for specific materials, systems and methods

We require information from authors about some types of materials, experimental systems and methods used in many studies. Here, indicate whether each material, system or method listed is relevant to your study. If you are not sure if a list item applies to your research, read the appropriate section before selecting a response.

Materials & experimental systems

n/a	Involved in the study
☐	☒ Antibodies
☐	☒ Eukaryotic cell lines
☒	☐ Palaeontology and archaeology
☒	☐ Animals and other organisms
☒	☐ Human research participants
☒	☐ Clinical data
☒	☐ Dual use research of concern

Methods

n/a	Involved in the study
☒	☐ ChIP-seq
☐	☒ Flow cytometry
☒	☐ MRI-based neuroimaging

Antibodies

Antibodies used	Anti-PARP1 (rabbit polyclonal, Cell Signalling Technology #9542), anti-OBFC1 (STN1, mouse monoclonal, Santa Cruz sc-376450), anti-53BP1 (rabbit polyclonal, NOVUS biologicals NB100-304), anti-Mad2L2/REV7 (rabbit monoclonal, Abcam ab180579), anti-Cas9 (mouse monoclonal, 7A9-3A3 Cell Signaling Technology), anti-Tubulin (mouse monoclonal, Sigma-Aldrich, T6199 clone DM1A), CF680 goat anti-rabbit IgG and CF770 goat anti-mouse IgG (Biotium)
Validation	The following antibodies were validated in knockout cells: anti-OBFC1 (STN1, mouse monoclonal, Santa Cruz sc-376450), anti-53BP1 (rabbit polyclonal, NOVUS biologicals NB100-304), anti-Mad2L2/REV7 (rabbit monoclonal, Abcam ab180579).<br><br>- Anti-PARP1 (rabbit polyclonal, Cell Signalling Technology #9542) validation stated on suppliers website: https://www.cellsignal.com/datasheet.jsp?productId=9542&images=1&protocol=0<br>- anti-OBFC1 (STN1, mouse monoclonal, Santa Cruz sc-376450) validation stated on suppliers website: https://datasheets.scbt.com/sc-376450.pdf<br>- anti-53BP1 (rabbit polyclonal, NOVUS biologicals NB100-304) validation stated on suppliers website: https://www.novusbio.com/products/53bp1-antibody_nb100-304#datasheet<br>- anti-Mad2L2/REV7 (rabbit monoclonal, Abcam ab180579) validation stated on suppliers website: https://www.abcam.com/mad2l2rev7-antibody-epr13657-ab180579.html

- anti-Cas9 (mouse monoclonal, 7A9-3A3 Cell Signaling Technology) validation stated on suppliers website: https://www.cellsignal.com/datasheet.jsp?productId=14697&images=1&protocol=0
- anti-Tubulin (mouse monoclonal, Sigma-Aldrich, T6199 clone DM1A) validation stated on suppliers website: https://www.sigmaaldrich.com/NL/en/product/sigma/t9026?gclid=EAIaIQobChMI4sa7wf--8QIVTeh3Ch0fTwI-EAAYASAAEgKt5vD_BwE#
- CF680 goat anti-rabbit IgG (Biotium) validation stated on suppliers website: https://biotium.com/product/goat-anti-rabbit-igg-hl-highly-cross-absorbed/?attribute_pa_conjugation=cf680
- CF770 goat anti-mouse IgG (Biotium) validation stated on suppliers website: https://biotium.com/product/goat-anti-rabbit-igg-hl-highly-cross-absorbed/?attribute_pa_conjugation=cf770

Eukaryotic cell lines

Policy information about cell lines

Cell line source(s)

- All mouse embryonic stem cell-lines are subclones of the 129/Ola-derived IB10 clone described in: Robanus-Maandag E, Dekker M, van der Valk M, Carrozza ML, Jeanny JC, Dannenberg JH, Berns A, te Riele H (1998) p107 is a suppressor of retinoblastoma development in pRb-deficient mice. Genes Dev 12: 1599–1609.
- mouse embryonic stem cells IB10 Polq-/- and Ku80-/- are previously described: Schimmel, J., Kool, H., van Schendel, R. & Tijsterman, M. Mutational signatures of non-homologous and polymerase theta-mediated end-joining in embryonic stem cells. EMBO J 36, 3634-3649, doi:10.15252/embj.201796948 (2017).
- Human RPE1-hTERT cells (ATCC (CRL-4000™)) deficient in p53 are described in: Benedict, B. et al. WAPL-Dependent Repair of Damaged DNA Replication Forks Underlies Oncogene-Induced Loss of Sister Chromatid Cohesion. Dev Cell 52, 683-698 e687, doi:10.1016/j.devcel.2020.01.024 (2020).

Cell-lines generated in this study:
- IB10 HPRT-GFP knockin clone 1
- IB10 HPRT-GFP knockin clone 2
- IB10 HPRT-GFP Polq-/- clone 1
- IB10 HPRT-GFP Polq-/- clone 2
- IB10 HPRT-GFP Ku80-/- clone 1
- IB10 HPRT-GFP Ku80-/- clone 2
- IB10 HPRT-GFP Ctc1-/- clone 1
- IB10 HPRT-GFP Ctc1-/- clone 2
- IB10 HPRT-GFP Stn1-/- clone 1
- IB10 HPRT-GFP Stn1-/- clone 2
- IB10 HPRT-GFP Polq-/- Ctc1-/- clone 1
- IB10 HPRT-GFP Polq-/- Ctc1-/- clone 2
- IB10 HPRT-GFP tp53bp1-/- clone 1
- IB10 HPRT-GFP tp53bp1-/- clone 2
- IB10 HPRT-GFP Shld2-/- clone 1
- IB10 HPRT-GFP Shld2-/- clone 2
- IB10 PrimPol-/- clone 1
- IB10 PrimPol-/- clone 2
- RPE1-hTERT p53-/- Rev7-/- clone 1
- RPE1-hTERT p53-/- Rev7-/- clone 2

Authentication

Human RPE1-hTERT cells were authenticated by STR profiling. Parental IB10 mouse embryonic stem cells were not validated. All knockout cells were validated by Western blot analysis and/or DNA sequencing.

Mycoplasma contamination

All cell lines were frequently tested negative for mycoplasma contamination.

Commonly misidentified lines
(See ICLAC register)

No commonly misidentified cell lines were used in this study

Flow Cytometry

Plots

Confirm that:

☒ The axis labels state the marker and fluorochrome used (e.g. CD4-FITC).

☒ The axis scales are clearly visible. Include numbers along axes only for bottom left plot of group (a 'group' is an analysis of identical markers).

☒ All plots are contour plots with outliers or pseudocolor plots.

☒ A numerical value for number of cells or percentage (with statistics) is provided.

Methodology

Sample preparation

Cells were trypsinized and resuspended in PBS with 2% BSA

Instrument	The NovoCyte NovosamplerPro (ACEA) was used to quatify eGFP and/or mCherry intensity. To sort eGFP negative cells, the BD FACSAria III (BD Biosciences) was used
Software	The NovoExpress and BD FACSDiva software was used to analyze the flow cytometry data
Cell population abundance	HPRT-eGFP mutant cells were selected by sorting 100.000 to 500.000 eGFP negative cells using a 85 uM nozzle. No purity check was performed
Gating strategy	Gates were defined by using untargeted wild-type (non HPRT-eGFP) and wild-type HPRT-eGFP control cells to discriminate between positive and negative eGFP expressing cells. Standard FSC/SSC gating was used to select for living single cells. An example of the gating strategy has been provided in the Source Data file

☒ Tick this box to confirm that a figure exemplifying the gating strategy is provided in the Supplementary Information.
